# Intergroup Conflict and Rational Decision Making

**DOI:** 10.1371/journal.pone.0114013

**Published:** 2014-12-02

**Authors:** Vicente Martínez-Tur, Vicente Peñarroja, Miguel A. Serrano, Vanesa Hidalgo, Carolina Moliner, Alicia Salvador, Adrián Alacreu-Crespo, Esther Gracia, Agustín Molina

**Affiliations:** 1 IDOCAL, University of Valencia, Valencia, Spain; 2 Department of Psychobiology, University of Valencia, Valencia, Spain; 3 Laboratory of Social Neuroscience, Department of Psychobiology and IDOCAL, University of Valencia, Valencia, Spain; Center of nonlinear, China

## Abstract

The literature has been relatively silent about post-conflict processes. However, understanding the way humans deal with post-conflict situations is a challenge in our societies. With this in mind, we focus the present study on the rationality of cooperative decision making after an intergroup conflict, i.e., the extent to which groups take advantage of post-conflict situations to obtain benefits from collaborating with the other group involved in the conflict. Based on dual-process theories of thinking and affect heuristic, we propose that intergroup conflict hinders the rationality of cooperative decision making. We also hypothesize that this rationality improves when groups are involved in an in-group deliberative discussion. Results of a laboratory experiment support the idea that intergroup conflict –associated with indicators of the activation of negative feelings (negative affect state and heart rate)– has a negative effect on the aforementioned rationality over time and on both group and individual decision making. Although intergroup conflict leads to sub-optimal decision making, rationality improves when groups and individuals subjected to intergroup conflict make decisions after an in-group deliberative discussion. Additionally, the increased rationality of the group decision making after the deliberative discussion is transferred to subsequent individual decision making.

## Introduction

“7^th^ Juror: So, what do we do now?

8^th^ Juror: Well, I guess we talk.”

*Twelve Angry Men*, by Reginald Rose (1955)

Intergroup conflict is a pervasive and ubiquitous phenomenon. The Institute for Economics & Peace has codified over 104,000 cases of terrorism in the world –and 64,000 people were killed– during the 10-year period from 2002 to 2011 [1]. Ethnic conflicts have taken millions and millions of lives since World War II [2]. For example, in Rwanda, the Hutu systematically killed their Tutsi neighbors with the explicit intention of exterminating them in 1994 [3]. In other regions of the world, continuous intergroup conflicts have been observed: Israelis vs. Palestinians, Catholics vs Protestants in Northern Ireland, Bosnia-Herzegovina vs. Croatia, etc. In addition, peace after conflict seems unstable [4], and people often have the feeling that the conflict can start up again at any time. Intergroup conflict is not new in humans. It has existed (and exists) across societies and over time. Refuting the argument that intergroup violence is a post-agricultural phenomenon, there is increasing evidence that hunter-gatherer societies were involved in wars and mass murder [5], [6]. This intergroup violence is also observed in chimpanzees, indicating a deep evolutionary history where intergroup conflict and aggression are present [7].

The pervasiveness of intergroup conflict is related to humans' high capacity to distinguish between in-group and out-group members [8]–[10]. This capacity is associated with our biological make-up and evolutionary history [11]–[14], and it underlies forces leading to both discrimination-aggression and cooperation-trust between groups. According to the male warrior hypothesis [15], males obtained reproductive and other benefits during our evolutionary past when they joined aggressive coalitions against members of out-groups. Females were also concerned about the out-group because contact with members of other groups increased the threat of sexual coercion and reduced the female mammalian mating strategy of reproductive choice [16]. Despite these forces toward distrust and discrimination of other groups, humans have also shown a capacity for cooperation and trust between groups. In an analysis of hominids during the 2.9-million-year Paleolithic time span, Kelly [17] described how friendly relationships between groups improved the use of a territory's resources and facilitated humans' expansion across the globe and the development of agriculture. Pinker [18] documented that intergroup violence has declined from pre-history to the present day as a result of historical forces, and that humans are equipped with motives that can orient them toward both distrust and cooperation. Wagner and Hewstone [19] also presented successful efforts to reconcile hostile groups in order to increase mutual trust and benefits after intergroup conflict. Finally, drawing on evolutionary game theory, scholars have highlighted the evolution of cooperation between groups, populations, and complex networks [20]–[23], illustrating several mechanisms through which cooperation is promoted or impaired [24].

The present study considers this paradoxical human attitude toward the out-group members by focusing attention on decision making and cooperation after intergroup conflict. Specifically, we examine the rationality of cooperative decision making, i.e. the extent to which groups take advantage of post-conflict situations to obtain opportunities for themselves from collaboration with the other group involved in the conflict. Although “The social - psychological literature is relatively mute about post-conflict processes” [25], the differentiation between affective and deliberative human thinking systems [26], [27] offers a theoretical basis associated with our evolutionary history for understanding the constraints and facilitators of cooperation after intergroup conflict. Post-conflict cooperation is fragile because hostility and negative emotional reactions toward the other group remain [28]. However, deliberative discussion can improve the rationality of the decision making [29] and increase the possibilities for mutual benefits. Building on this logic, we suggest that, although recovery after intergroup conflict is difficult, deliberative efforts can help to offer a chance for rational cooperation and mutual benefits between groups.

### Intergroup Conflict and Rationality of Decision Making

Scholars have revealed the existence of obstacles in recovering the relations after intergroup conflict because of psychological wounds produced during the conflict [28], [30]. Negative feelings associated with conflicts provoke sub-optimal or irrational decision making and result in a loss of opportunities. Groups do not seem to be able to take advantage of cooperation with other groups for their own benefit. The role of affect in decision making offers an explanation for this phenomenon. Despite the traditional emphasis on cognitive aspects of decision making, affect is increasingly considered by decision researchers. Zajonc's [31] seminal work emphasized the importance of affect as an automatic response guiding subsequent information processing and decision making. The *dual-process theories* of thinking also reinforce the idea that, in addition to deliberative or cognitive information processing, humans use an automatic experiential system with an affective base [26]. Slovic and colleagues [32], [33] considered these antecedents when they proposed the *affect heuristic*. Heuristics make it possible to deal with complex problems in life through approximation and by-passing more deliberative analyses [34], [35]. The affect heuristic assumes that humans make decisions based on positive and negative feelings that are consciously and unconsciously associated with their representations of objects and events [33], providing an immediate positive or negative evaluation of stimuli and a quick response in the decision making.

All of these theoretical considerations are congruent with Damasio's [36] hypotheses about somatic markers and rational behavior. Lifetime learning associates positive and negative feelings with representations that become *marked* and are connected directly or indirectly to somatic states. Positive markers associated with image outcomes stimulate incentives and motivation, while negative markers related to image outcomes produce alerts. Damasio suggested that these somatic markers allow humans to respond quickly and efficiently. In fact, a lack of markers, observed in individuals with some types of brain damage, hinders the rationality of decision making [37]. Thus, the affect heuristic, which is also present in other primates [38], is adaptive and facilitates humans' navigation in an uncertain world, leading Finucane et al. [32] to state that it, “can be far easier – more efficient – than weighing the pros and cons or retrieving from memory many relevant examples, especially when the required judgment or decision is complex or mental resources are limited”. However, this evolutionary adaptive strategy has costs. In some relevant cases, there are discrepancies between the experiential (intimately associated with affect, holistic, rapid, and with a long evolutionary history) and deliberative (relatively affect-free, analytical, relatively slow, and with a brief evolutionary history) systems described in the dual-process theories [26], [27]. The experiential and affective system can guide the behavior in one direction, while the deliberative system can guide the behavior in the opposite way. Hine et al. [39] observed that users of wood heaters presented more positive affective associations with wood heating and a less rational analysis of risk than non-users. Their data also suggested that, when there is a gap, affective associations predominate over deliberative analyses in guiding individual behavior. Another example of failure of the experiential system is linked to the smoking behavior. Slovic [40] argued that young smokers enjoy smoking because they find it new and exciting, without performing a deliberative analysis of the risks of nicotine dependence over time.

In consonance with these arguments, emotions can play a role in decision making after intergroup conflict. It is well known that conflict and emotions are inextricably connected [41]. Conflict is manifested only when individuals are emotionally charged [42]. Negative feelings associated with out-group members because of a conflict situation can interfere with rational decision making and cooperation after the conflict. Decision making is emotionally driven against cooperation with members of the out-group, and individuals can even have difficulties in taking advantage of good opportunities for their own interests if the decision also produces benefits for members of the out-group. Accordingly, we propose the following:

#### Hypothesis 1

Intergroup conflict has a negative effect on the rationality of cooperative decision making, so that individuals and groups subjected to intergroup conflict are less able, compared to those not subjected to intergroup conflict, to make optimal decisions that produce opportunities for themselves stemming from cooperation with members of the out-group.

### In-group Deliberative Discussion

One way to deal with a lack of rational decision making after conflict and achieve a more positive and cooperative approach to post-conflict situations is to create a social context where deliberative efforts and information processing are stimulated. Kugler et al. [29] reviewed the literature about the decision making of individuals and groups. They observed that groups are more rational in their decision making than individuals are, especially when information has to be processed in order to understand the structure and rules of the task. Kugler and colleagues argued that the group offers a better view of the problem, and the interaction among group members provides more information processing capabilities and allows the correction, through discussion, of groups' errors. Maciejovsky et al. [43] confirmed not only that groups are more rational than individuals, but also that there is a transference from groups to individuals. Participation in group decision making increases the subsequent decision making quality of individuals.

In the present study, we extend the investigation of deliberative group discussion to intergroup conflicts. We propose that when groups have the opportunity to make a deliberative analysis, opportunities for rational decision making and mutual benefits increase, and this rational cooperation is transferred to individuals. This *civilizing* role of groups is also part of our evolutionary history. Scholars assume that early hominids increased their performance when group members communicated with each other about dispersed food sources [44]. Evidence suggests that cooperation, social bonds and social learning within groups of primates improve the competitive success and reproductive performance of individuals [45]. Boehm et al. [46] documented that tribes usually organize group discussions about important decisions, such as whether or not to attack a neighboring tribe, where the authority (the “big man”) helps as the chairman of the meeting rather than as an authoritarian dictator. Of course, the group can be irrational. For example, lack of critical analysis and conformism with authority (e.g., groupthink) can take place under some circumstances [47]. However, if group discussion is reinforced, the deliberative system has a chance to prosper. In post-conflict situations, deliberative group analyses can reorient the potential failures of the experiential system, pointing out benefits for in-group members –even when they also involve cooperation and benefits for the other group– and transferring this higher decision making rationality to individual members. In sum, we propose the following:

#### Hypothesis 2a

After in-group discussion, the rationality of the cooperative decision making of groups and individuals subjected to intergroup conflict improves, so that groups and individuals are more capable of making optimal decisions that can produce opportunities for them stemming from cooperation with members of the out-group.

#### Hypothesis 2b

After in-group discussion, the decision making of groups is transferred to the individual decision making of their members, so that there is a positive relationship between the rationality of cooperative group decision making and the rationality of subsequent cooperative individual decision making.

In summary, the present study investigates whether intergroup conflict hinders the rationality of individual and cooperative decision making, as well as whether in-group deliberative discussion improves rationality. We were able to show that intergroup conflict led to sub-optimal decision making and rationality improved when groups and individuals subjected to intergroup conflict make decisions after an in-group deliberative discussion.

## Method

### Participants

After on-line medical screening –exclusion criteria were significant medical or psychiatric illness, medication, smoking more than 10 cigarettes per day, and alcohol and/or drug abuse–, a total of 141 healthy university students (*M_age_* = 21.61, *SD* = 3.14; 54.6% women) voluntarily participated in the experiment. During recruitment, we told participants they would receive an undetermined amount of money for participating in the experiment. When all the sessions had ended, we informed participants about the logic of the experiment, and all of them received €9 (about 12 USD) for their participation.

### Procedure

The experiment had three sequential decision-making stages: (a) individual, (b) group, and (c) individual. We tested whether intergroup conflict impacts the rationality of decision making oriented toward individuals' cooperation (seeking opportunities for themselves from decisions that also produce benefits for the other group) after the conflict is over (a), and whether this effect on rationality remains over time and in group (b) and individual (c) decision making. Additionally, we examined whether group decision making (b) improves the level of rationality, and to what extent this change in the rationality of group decision making is transferred to subsequent individual decision making (c). Of the total number of participants, 84 (50% women) were randomly assigned to the experimental condition, participating in sessions with six individuals of the same sex. The intergroup conflict was simulated by using the task called “Viking Investments”, with the permission of its creators [48]. This task describes a complex and multifaceted conflict between a real estate investment company and a carpentry business. Howard et al. [49] used this task to study intergroup disputes. We followed the same procedure. Thus in each session, three of the participants randomly represented the real estate investment company, while the other three were representatives of the carpentry business. Each of the two parties in the conflict received a different document describing the conflict. The information induced each party to think that the other party was responsible for the problems caused. After an individual reading of the document (35 min), each group of three (28 groups) independently prepared a discussion meeting with the other group (20 min). The face to face interaction (14 interactions or sessions) between the two groups lasted 10 min. Given the complexity and multifaceted nature of the dispute, the time allotted for the face to face interaction only allowed participants to become more aware of the intergroup conflict and the different perspectives of the two groups. Immediately after the meeting, one of the researchers thanked the participants and informed them that the conflict was over.

The participants' next task was to engage in a trust game variant that we created to register the rationality of their cooperative decision making after the intergroup conflict. In the traditional version of this game [50], the Trustor (individual or group) receives an initial endowment and decides how much of the endowment is sent to the Trustee (individual or group). The amount is then multiplied by a known factor (often tripled) en route from the Trustor to the Trustee. After tripling, the Trustee decides what amount is to be sent to the Trustor as an act of reciprocity. In our experiment, we only focused on sending the money. For each participant, we assigned an initial €3 (about 4 USD) endowment. In the first stage (a), after the face to face interaction with the other group, each participant decided, anonymously and individually, what amount of money he/she would send to a member of the other group, with an explicit indication that the member of the other party would triple the amount, but with the obligation to return at least the original amount to the individual sender. The most rational decision is to send the total amount (€3) because there is no risk (the original amount is guaranteed to the sender), and it increases resources, facilitating additional reciprocation. However, it requires cooperation with a group with which the participant in question has had a conflict, previously simulated. In the second stage of the experiment, participants were grouped again. The original groups were asked to replace the previous individual decisions with a group and consensual decision (b) to represent all the in-group members. Each group met and deliberated for 5 min to make the decision. After this group decision, participants had one last chance to reconsider their decision (third stage). They were asked to make this final decision individually and anonymously (c).

Participants assigned to the control condition were involved in the same process. Of the 60 people convened, three of them indicated at the last minute that they could not participate due to unexpected events unrelated to the experiment. Thus, 57 participants (61.4% women) were in the control condition, and three groups were composed of two members (20 groups and 10 interactions). The only difference, with respect to the experimental condition, was lack of conflict. After the individual reading (35 min), each group was asked to prepare a summary of the document (Viking Investments), using a standardized sheet (20 min), in order to communicate this information to the other group in the face to face interaction (10 min). The three stages of decision making –individual (a), group (b), individual (c)– were also implemented.

As discussed below, self-report and heart rate data were also measured during the experiment. All sessions were held in the same laboratory at the university. We carried out a pilot study to adjust the duration of each phase of the experiment and check that participants understood the instructions properly. Two experimenters monitored each experimental session and used a chronometer to control the timing of the session. The experimenters were always the same across sessions. Participants were asked to refrain from eating, smoking and consuming caffeine during the two hours before the laboratory session. They were also instructed to refrain from intense physical exertion for at least 48 hours prior to the laboratory session and not sleep less than usual (seven to eight hours). The experimenters checked whether they had followed these instructions.

### Ethics statement

The study was conducted in accordance with the Declaration of Helsinki, and the protocol and conduct were approved by the Ethics Committee in experimental research of the University of Valencia. The president of the ethics committee was Dr. Màrius V. Fuentes Ferrer (University of Valencia). All participants gave written and informed consent to the experimental procedure.

## Results

### Preliminary analyses

We carried out preliminary analyses to confirm that our intergroup conflict experimental condition had an impact on perceptual measures of conflict, affect state, and heart rate. At the end of the experiment, participants indicated the level of task (derived from activities or tasks) and relational (derived from personal issues or values) conflict they perceived during the face-to-face interaction with the other group. To this end, we used the questionnaire by De Dreu et al. [51], adapted to the intergroup situation. The Cronbach Alphas were.91 and.87 for task and relational conflicts, respectively. Our findings show that perceptions of task (*t*_(93.74)_  = 9.49; *p*<.01) and relational (*t*_(139)_  = 6.85; *p*<.01) conflict were higher among participants in the experimental condition than among those in the control condition. In contrast, there were no significant differences between the two parties of the conflict (real estate investments vs. carpentry business) in the experimental condition on their perceptions of task (*p* = .14) and relational conflict (*p* = .27). Participants also reported on their affect states at three measurement times: when they arrived at the experimental site; after the group preparation of the discussion meeting; and at the end of the experiment. We used the well-known PANAS (The Positive and Negative Affect Schedule) instrument to provide independent indexes of their positive (10 items, the Cronbach Alpha was.81) and negative (10 items, the Cronbach Alpha was.78) affect states [52]. There was a significant interaction effect between the conditions and the moment of measuring positive affect (*F*_(2, 276)_  = 3.37; *p*<.05), as well as for the moment of measuring negative affect (*F*_(2, 276)_  = 14.74; *p*<.01). Bonferroni *post hoc* tests revealed that there were no significant differences between experimental and control participants in their positive (*p* = .66) and negative (*p* = .88) affect states before the experiment. When they were in the *core* of the conflict (after the preparation of the discussion meeting), participants in the experimental condition (Mean ±SE = 2.06±0.07) presented higher negative affect state than participants in the control condition (Mean ±SE = 1.54±0.08) (*p*<.01), while no significant difference was found in their positive affect state (*p* = .38). At the end of the experiment, when participants had participated in the group decision and made the final individual decision, the positive affect state was higher among participants in the experimental condition (Mean ±SE  = 3.24±0.08) than among those in the control condition (Mean ±SE = 2.94±0.10) (*p*<.05).

We also assessed participants' heart rate. Heart rate is indicative of a visceral emotional arousal produced by the autonomic network [53]. Previous research studies have found links from conflict to heart rate [54], especially in family conflicts. We extend this analysis to intergroup conflict. Heart rate was measured by means of a Polar©RS800cx watch (Polar CIC, USA), which consists of a chest belt for the detection and transmission of heartbeats and a watch for data storage. The watch records R-R intervals with a sampling frequency of 1000 Hz. Data were analyzed using HRV Kubios Analysis software (Biomedical Signal Analysis Group, University of Kuopio, Finland). Before the experiment (when participants arrived at the laboratory area), there were no significant differences between the control and experimental conditions (*t*_(82)_  = 1.04; *p* = 0.30). In contrast, our results corroborated that heart rate was higher during the core of the intergroup conflict (preparation of the meeting and face to face interaction) among participants in the experimental condition than among those in the control condition (*t*_(86)_  = 2.45, *p*<.05 and *t*_(75.21)_  = 3.57, p<.01, respectively).

All of these preliminary results confirmed that the experimental condition worked. The simulation of intergroup conflict was able to increase perceptions of conflict and activate negative feelings (negative affect state and heart rate).

### Testing of Hypotheses

**Figure 1:**
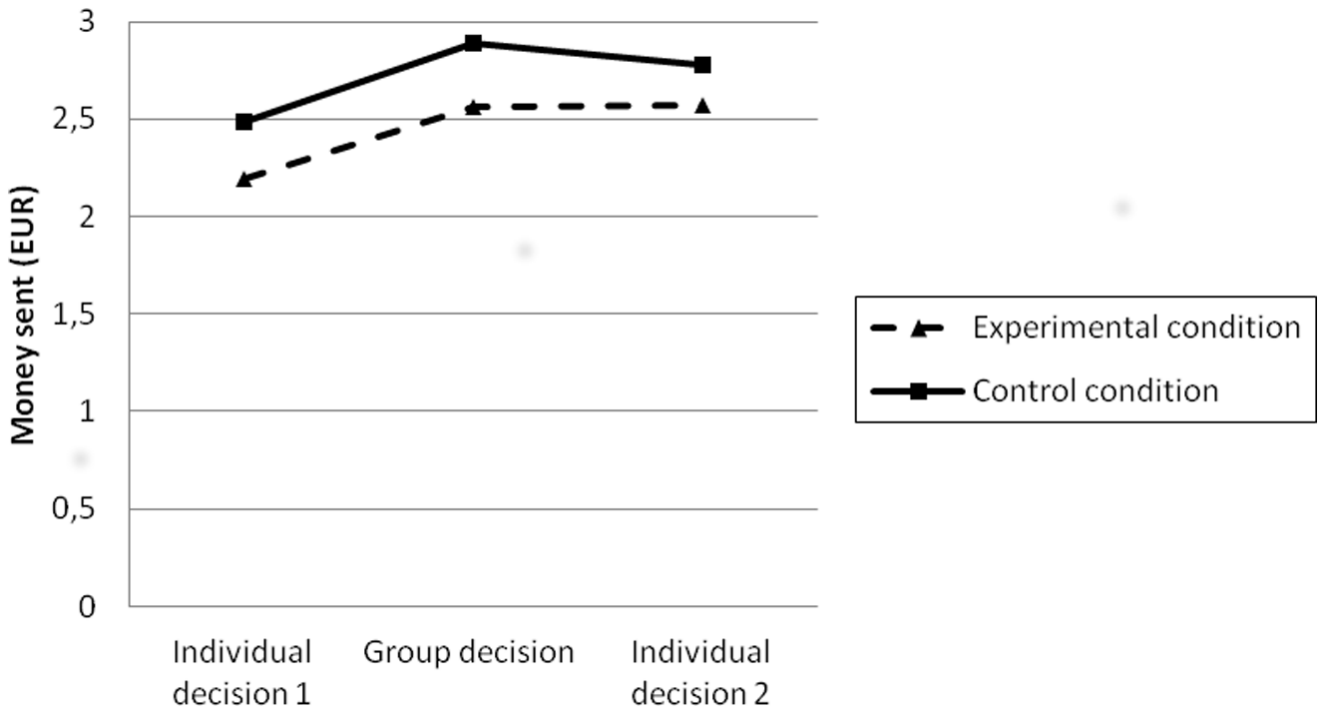
Three stages in decision making. Each line represents the average money sent (from 0 to 3 Euros) in the three decision-making stages in the experimental and control conditions. The first stage (a) corresponds to an individual decision, the second (b) to a group decision, and the third (c) to an individual decision. In each of the two individual decisions, the average amount sent by each participant individually to the out-group is represented. In the group decision, the average amount sent by each group to the other group is represented. 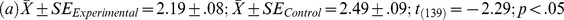 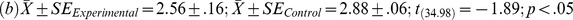 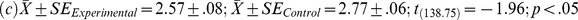

The first step in our analyses was to find out whether the intergroup conflict reduced the rationality of cooperative decision making (H1). Immediately after the conflict simulation –first individual decision making (a)–, we confirmed that participants in the experimental condition sent less money to the out-group members than participants in the control condition (*t*_(139)_  = −2.29; *p*<.05). We also observed that this difference remained over time in the subsequent decision making at the group (b) and individual (c) levels (see Figure 1). Participants subjected to intergroup conflict sent less money to the other group than participants in the control condition, both in the group (b) (*t*_(34.98)_  = −1.89; *p*<.05) and final individual (c) (*t*_(138.75)_  = −1.96; *p*<.05) decision making. In contrast, we did not observe statistically significant differences between men and women at any of the three time points: (a), (b), and (c) (all *p*>.05). As expected, intergroup conflict hinders the rationality of cooperative decision making.

The second step in the analyses was to find out whether group discussion increases the rationality of cooperative decision making (H2a). Concentrating our attention on the experimental condition (intergroup conflict), we found that the change from the first individual decision making (a) to the group decision making (b) was statistically significant (*t*_(83)_  = −3.53; *p*<.01). Thus, the money sent to out-group members increased significantly after the deliberative meeting where they agreed on the amount to be sent by the group as a whole. We also observed that the change from the first individual decision making (a) to the final individual decision making (c) was statistically significant (*t*_(83)_  = −3.90; *p*<.01). Participants sent more money in the definitive individual decision making (c) than in the initial one (a). In contrast, the change from the group decision making (b) to the final individual decision making (c) was not significant. In general, we confirmed that the rationality of cooperative decision making increases after the in-group deliberative discussion.

The final step in our analyses focused on the final individual decision making (c) of participants subjected to the intergroup conflict simulation (experimental condition). To test H2b, we examined the links from the first individual decision making (a) and the group decision making (b) to the final individual decision making (c). To this end, we ran a hierarchical linear modeling (HLM) analysis using LISREL 8.80 [55]. HLM allows the simultaneous examination of the relationships between variables at different levels of analysis (individual- and group-level decisions) [56]. Before testing the links from the individual-level (a) and group-level (b) decisions to the final individual-level decision (c), we conducted a null model for the dependent variable, which is a requirement for cross-level analyses [57], [58]. This preliminary step is designed to assess the systematic within- and between-unit variance in the dependent variable. Results of the null model –without predictors– revealed that 39.12% of the total variance in the final individual-level decision (c) (τ_00_ = .230, σ_2_ = .358, *χ^2^* = 182.14, *df* = 3, *p*<.001) was due to belonging to the group. Consistent with the multilevel nature of the data, the intercept term of the dependent variable varied significantly across groups, allowing the subsequent computation of cross-level analyses. As depicted in Table 1, the results of the cross-level model –with predictors– showed that when testing the relationship of both variables (e.g., first individual decision and group decision), only the group decision (b) was significantly related to the final individual decision (c) (*γ* = .68, *p*<.01). While the association with the group decision making was strong, the link with the first individual decision making was not significant. Thus, our findings corroborated that the final individual decision (c) of participants subjected to intergroup conflict is especially related to the decision previously made by the group to which each participant belonged (b). Group deliberative discussion has an effect on rational decisions made by the individual and by the group.

**Table 1:** Hierarchical linear modeling (HLM) results.

		Individual decisión (c)	
		Null model	Model with predictors
Level 1 (*n* = 84)	Intercept	2.57** (.11)	2.62** (.12)
	Individual decision (a)	–	.06 (.11)
**Level 2 (n = 28)**	**Group decision (b)**	–	.68** (.21)
**Within-unit variance (σ2)**		.358	.345
**Between-unit variance (τ00)**		.230	.098
**Within-unit ** ***R2***		–	3.63%
**Between-unit ** ***R2***		–	57.39%
**Number of free parameters (** ***df*** **)**		3	10
**Model deviance (χ2)**		182.14	168.34
**Δ** ***df***		–	7
**Δχ2**		–	13.80

Standard errors are reported in parentheses.

**p<.001.

### Post-experimental qualitative insights

Once all the experimental sessions had ended, we had a meeting with the participants to explain the logic of the experiment. In addition, we explored their opinions about decisions required in the experiment. About 40% of the women and 40% of the men subjected to intergroup conflict made the optimal decision: they sent the total amount to the out-group members. They used the words “surprise” and “incomprehensible decision” to describe the decision to send less than the €3 decided on by other individuals. The rest of the participants recognized that the optimal decision was to send the €3, but they indicated that they “could not in any way” give the total amount to the members of the other group. We also explored the in-group deliberative discussion, corroborating the positive effect of this discussion. For example, one of the participants indicated that the discussion “made us aware that there were better alternatives in the decision about sending the money”.

## Discussion

We examined the impact of intergroup conflict on the rationality of cooperative decision making. Our findings showed that intergroup conflict facilitates sub-optimal decision making, reducing the opportunities to benefit from cooperation with out-group members. Rational cooperation, however, improved when groups and individuals made decisions after an in-group deliberative discussion. Additionally, after the deliberative discussion, the rationality of the group decision making was transferred to subsequent individual decision making.

The literature has shown a paradoxical attitude of humans with respect to the relations between groups. On the one hand, aggression and distrust have been seen as adaptive because during our evolutionary history they provided benefits for both men [15] and women [16]. On the other hand, cooperation between groups has been considered a requirement for the survival and expansion of humans in the world [17]. The present experiment contributes to clarifying how decision making operates in the relations between groups. We focused on the obstacles to rational cooperation presented by groups that have experienced intergroup conflicts. More specifically, we wondered why these groups find it difficult to make decisions for their own benefit stemming from cooperation with other groups. We also explored a specific way to improve the rational decision making of these groups and their members after an intergroup conflict: the in-group discussion. Taken together, our findings contribute to understanding post-conflict processes, a research area that has been relatively neglected by the social - psychological literature [25], in spite of its importance in decision making and intergroup cooperation.

In general terms, our results indicate that, after an intergroup conflict, rationality in decision making suffers. Groups subjected to intergroup conflict take less advantage of cooperation to benefit themselves than groups not subjected to intergroup conflict. This effect was persistent over time and observed in both individual and group decision making. We interpret these findings based on the differentiation between deliberative vs. experiential systems in decision making [26] and the affect heuristic [33]. Almost inevitably, intergroup conflict is emotionally associated with a state of alert that has an influence on subsequent decision making. In fact, compared to participants in the control condition, those in the experimental condition indicated a higher negative affect state during the core of the conflict and presented a higher heart rate. In a number of situations, the emotionally driven decision is adaptive and permits a rational and efficient performance. It facilitates a type of learning that is necessary in order to make rational decisions in our lives because events and objects are consciously and unconsciously associated with emotions, permitting rapid and adequate responses [36]. For example, in post-conflict situations, humans can respond to the potential dangers coming from another group quickly because the out-group members are associated with negative emotional experiences from previous conflicts. However, the experiential system can produce failures [38], [39], as we observed in the current study. After an intergroup conflict, emotions can interfere with the perception of benefits and opportunities that can be obtained through cooperation with members of the out-group. In our experiment, sending the total amount means cooperating with members of the out-group (they triple), but this decision is optimal because it increases opportunities for in-group members without taking risks (the original amount is assured). Transferred to real life situations, this phenomenon can partly explain difficulties in reconciliation, even when cooperation involves mutual benefits and cost avoidance (e.g., in terms of loss of money and/or human lives).

Our evolutionary history, however, has equipped humans with a deliberative system that can compensate, to some degree, for the failures of automatic responses [34]. The deliberative system is analytical and relatively slow. One way to stimulate this system is through group discussion. As we mentioned earlier, when group members contribute to the decision making with their information processing capabilities, better analysis and error correction are possible [29]. Our findings are congruent with this argument: the money sent increased significantly from the first individual decision making (after the conflict) to both the group and individual decisions made after a deliberative discussion that required the consensus of its members.

In consonance with previous research efforts [43], the group can also play a *civilizing* role, directing individual behavior toward higher levels of rational cooperation. In our cross-level analysis, the final individual decision presented a strong relationship with the decision previously made by the in-group in question, while there was no relationship with the first individual decision made before the in-group discussion. Thus, the idea that the individual decision making is changed was supported by the present experiment. The group, as part of the social context, can have a negative influence on the individual, for example, in terms of groupthink [47]. However, we observed positive effects when discussion is stimulated, and the individual may become aware of other alternatives that are more beneficial to his/her interests even if they require cooperation with members of the out-group involved in a previous conflict. In sum, group deliberative discussion can help individuals to reconsider their decisions and better analyze the pros and cons of different alternatives, leading individuals and groups to make more rational decisions.

## Conclusions

The affect heuristic and the experiential approach to decision making are rational in many situations. They make it possible to deal with environmental requirements at minimal cost. However, rationality decreases when automatic emotions are not able to anticipate the consequences of decisions adequately. In the current experiment, intergroup conflict –and the associated negative emotions– hinders the perception of opportunities to satisfy their own interests through cooperation with out-group members. Group deliberative discussion serves to correct this failure, at least in part, indicating that the deliberative system and the sophisticated social nature of humans play a critical role in our rationality and in our way of managing relevant challenges. Although the scientific study of rationality is in its infancy [59], it seems that experiential and deliberative systems of thinking, in both individual and group decision making, help humans to navigate in a complex world where demands sometimes exceed their capabilities. One relevant goal is to define how and when each of the two systems produces better decisions about crucial issues such as the intergroup conflicts investigated in the present study.

## Supporting Information

Database S1 **Data base description**. Condition: 0 =  Experimental condition; 1 =  Control condition. Conflict role: A =  real estate investment company; B =  carpentry business. Gender: 0 =  Male; 1 =  Female. Taskconflict: perception of task conflict. Relconflict: Perception of relationship conflict. PANAS1_positive: Positive affect state before the experiment. PANAS2_positive: Positive affect state in the core of the conflict. PANAS3_positive: Positive affect state at the end of the experiment. PANAS1_negative: Negative affect state before the experiment. PANAS2_negative: Negative affect state in the core of the conflict. PANAS3_negative: Negative affect state at the end of the experiment. HR_before: Heart rate before the experiment. HR_meeting: Heart rate during the preparation of the meeting. HR_interaction: Heart rate during the face to face interaction.
(XLS)

## References

[1] The Institute for Economics & Peace website (2012) Global Terrorism Index Report. Available: http://economicsandpeace.org/wp-content/uploads/2011/09/2012-Global-Terrorism-Index-Report.pdf. Accessed 2013 Nov 25.

[2] Horowitz DL (1985) Ethnic groups in conflict. Berkeley, CA: University of California Press.

[3] Fujii LA (2010) Shades of truth and lies: Interpreting testimonies of war and violence. J Peace Res 47:231–241.

[4] Collier P (2009) Wars, guns, and votes: Democracy in dangerous places. London, England: Bodley Head.

[5] David Adams' website (2008) The history of the culture of war. Available: http://culture-of-peace.info/books/history/culture.html Accessed 2013 Dec 3.

[6] Walker PL (2001) A bioarchaeological perspective on the history of violence. Annu Rev Anthropol 30:573–596.

[7] Sherrow HM (2012) Violence across animals and within early hominids. In: Shackelford TK, Weekes-Shackelford VAeditors.The oxford handbook of evolutionary perspectives on violence, homicide, and war. New York: Oxford University Press. pp.23–40.

[8] Allport GW (1954) The nature of prejudice. Addison-Wesley: Cambridge.

[9] Brewer MB (1999) The psychology of prejudice: Ingroup love or outgroup hate? J Soc Issues 55:429–444.

[10] Tajfel H, Turner JC (1986) The social identity theory of intergroup behavior. In: Worchel S, Austin WGeditors.Psychology of intergroup relations. Chicago, IL: Nelson-Hall. pp.7–24.

[11] De Dreu CKW (2012) Oxytocin modulates cooperation within and competition between groups: An integrative review and research agenda. Horm Behav 61:419–428. 22227278 10.1016/j.yhbeh.2011.12.009

[12] De Dreu CKW, Shalvi S, Greer LL, Van Kleef GA, Handgraaf MJJ (2012) Oxytocin motivates non-cooperation in intergroup conflict to protect vulnerable in-group members. PLoS ONE 7:1–7. 10.1371/journal.pone.0046751 PMC3492361 23144787

[13] De Dreu CKW, Greer LL, Handgraaf MJJ, Shalvi S, Van Kleef GA, et al (2010) The neuropeptide oxytocin regulates parochial altruism in intergroup conflict among humans. Science 328:1408–1411. 20538951 10.1126/science.1189047

[14] Yamagishi T, Mifune N (2009) Social exchange and solidarity: In-group love or out-group hate? Evol Hum Behav 30:229–237.

[15] Van Vugt M, De Cremer D, Janssen DP (2007) Gender differences in cooperation and competition: The male-warrior hypothesis. Psychol Sci 18:19–23. 17362372 10.1111/j.1467-9280.2007.01842.x

[16] McDonald MM, Navarrete CD, Van Vugt M (2012) Evolution and the psychology of intergroup conflict: The male warrior hypothesis. Philos Trans R Soc Lond B Biol Sci 367:670–679. 22271783 10.1098/rstb.2011.0301 PMC3260849

[17] Kelly RC (2004) The evolution of lethal intergroup violence. PNAS 102:15294–15298. 10.1073/pnas.0505955102 PMC1266108 16129826

[18] Pinker S (2011) The better angels of our nature: Why violence has declined. Viking: New York.

[19] Wagner U, Hewstone M (2012) Intergroup contact. In: Tropp LReditor.The oxford handbook of intergroup conflict. New York, NY: Oxford University Press. pp.193–209.

[20] Jiang L-L, Perc M (2013) Spreading of cooperative behavior across interdependent groups. Sci Rep 3:2483. 23963495 10.1038/srep02483 PMC3748424

[21] Perc M, Gómez-Gardeñes J, Szolnoki A, Floría LM, Moreno Y (2013) Evolutionary dynamics of group interactions on structured populations: A review. J R Soc Interface 10:20120997. 23303223 10.1098/rsif.2012.0997 PMC3565747

[22] Wang Z, Szolnoki A, Perc M (2014) Rewarding evolutionary fitness with links between populations promotes cooperation. J Theor Biol 349:50–56. 24508726 10.1016/j.jtbi.2014.01.037

[23] Wang Z, Xia C-Y, Meloni S, Zhou C, Moreno Y (2013) Impact of social punishment on cooperative behavior in complex networks. Sci Rep 3:3055. 24162105 10.1038/srep03055 PMC3808815

[24] Novak MA (2006) Five rules for the evolution of cooperation. Science 314:1560–1563. 17158317 10.1126/science.1133755 PMC3279745

[25] De Dreu CKW (2010) Social conflict: The emergence and consequences of struggle and negotiation. In: Fiske ST, Gilbert DT, Lindzey Geditors.Handbook of social psychology.Hoboken, NJ: Wiley. pp.983–1024.

[26] Epstein S (1994) Integration of the cognitive and psychodynamic unconscious. Am Psychol 49:709–724. 8092614 10.1037//0003-066x.49.8.709

[27] Pacini R, Epstein S (1999) The relation of rational and experiential information processing styles to personality, basic beliefs, and the ratio-bias phenomenon. J Pers Soc Psychol 76:972–987. 10402681 10.1037//0022-3514.76.6.972

[28] Al Ramiah A, Hewstone M (2013) Intergroup contact as a tool for reducing, resolving, and preventing intergroup conflict: Evidence, limitations, and potential. Am Psychol 68:527–542. 24128316 10.1037/a0032603

[29] Kugler T, Kausel EE, Kocher MG (2012) Are groups more rational than individuals? A review of interactive decision making in groups. Wiley Interdiscip Rev Cogn Sci 3:471–482. 26301530 10.1002/wcs.1184

[30] Staub E (2006) Reconciliation after genocide, mass killing, or intractable conflict: Understanding the roots of violence, psychological recovery, and the steps toward a general theory. Polit Psychol 27:867–894.

[31] Zajonc RB (1980) Feeling and thinking: Preferences need no inferences. Am Psychol 35:151–175.

[32] Finucane ML, Alhakami A, Slovic P, Johnson SM (2000) The affect heuristic in judgments of risks and benefits. J Behav Decis Mak 13:1–17.

[33] Slovic P, Finucane ML, Peters E, MacGregor DG (2002) The affect heuristic. In: Gilovich T, Griffin D, Kahneman Deditors.Heuristics and biases: The psychology of intuitive judgment. New York: Cambridge University Press. pp.397–420.

[34] Kahneman D (2011) Thinking, fast and slow. New York: Farrar, Straus, and Giroux.

[35] Kahneman D (2003) A perspective on judgment and choice: Mapping bounded rationality. Am Psychol 58:697–720. 14584987 10.1037/0003-066X.58.9.697

[36] Damasio AR (1994) Descartes' error: Emotion, reason, and the human brain. New York: Avon.

[37] Damasio AR, Tranel D, Damasio HC (1990) Individuals with sociopathic behavior caused by frontal damage fail to respond autonomically to social stimuli. Behav Brain Res 41:81–94. 2288668 10.1016/0166-4328(90)90144-4

[38] Kralik JD, Xu ER, Knight EJ, Khan SA, Levine WJ (2012) When less is more: Evolutionary origins of the affect heuristic. PLoS ONE 7:e46240. 23056270 10.1371/journal.pone.0046240 PMC3463577

[39] Hine DW, Marks ADG, Nachreiner M, Gifford R, Heath Y (2007) Keeping the home fires burning: The affect heuristic and wood smoke pollution. J Environ Psychol 27:26–32.

[40] Slovic P (2001) Cigarette smokers: Rational actors or rational fools? In: Slovic Peditor.Smoking: Risk, perception, and policy.Thousand Oaks, CA: Sage. pp.97–124.

[41] Nair N (2007) Towards understanding the role of emotions in conflict: A review and future directions. International Journal of Conflict Management 19:359–381.

[42] Bodtker AM, Jameson JK (2001) Emotion in conflict formation and its transformation: application to organizational conflict management. International Journal of Conflict Management 12:259–275.

[43] Maciejovsky B, Sutter M, Budescu DV, Bernau P (2013) Teams make you smarter: How exposure to teams improves individual decisions in probability and reasoning tasks. Manage Sci 59:1255–1270.

[44] Sumner S, King AJ (2011) Actions speak louder than words in socially foraging human groups. Commun Integr Biol 4:755–757 Available: http://www.ncbi.nlm.nih.gov/pmc/articles/PMC3306351/. 22446547 10.4161/cib.17701 PMC3306351

[45] Silk JB (2007) Social components of fitness in primate groups. Sci 317:1347–1351. 10.1126/science.1140734 17823344

[46] Boehm C, Antweiler C, Eibl-Eibesfeldt I, Kent S, Knauft BM, et al (1996) Emergency decisions, cultural selection mechanics, and group selection. Curr Anthropol 37:763–793.

[47] Janis IL (1981) Groupthink. Boston: Houghton Mifflin.

[48] Greenhalgh L (1993) Viking investments. Hanover, NH: Creative Consensus Inc.

[49] Howard ES, Gardner WL, Thompson L (2007) The role of the self-concept and the social context in determining the behavior of power holders: Self-construal in intergroup versus dyadic dispute resolution negotiations. J Pers Soc Psychol 93:614–631. 17892335 10.1037/0022-3514.93.4.614

[50] Song F (2009) Intergroup trust and reciprocity in strategic interactions: Effects of group decision-making mechanisms. Organ Behav Hum Decis Process 108:164–173.

[51] De Dreu CKW, Evers A, Beersma B, Kluwer ES, Nauta A (2001) A theory-based measure of conflict management strategies in the work place. J Organ Behav 22:645–668.

[52] Watson D, Clark LA, Tellegen A (1988) Development and validation of brief measures of positive and negative affect: The PANAS scales. J Pers Soc Psychol 54:1063–1070. 3397865 10.1037//0022-3514.54.6.1063

[53] Yang TT, Simmons AN, Matthews SC, Tapert SF, Bischoff-Grethe A, et al (2007) Increased amygdala activation is related to heart rate during emotion processing in adolescent subjects. Neurosci Lett 428:109–114. 18029095 10.1016/j.neulet.2007.09.039 PMC2171034

[54] Newton TL, Sanford JM (2003) Conflict structure moderates associations between cardiovascular reactivity and negative marital interaction. Health Psychol 22:270–278. 12790254 10.1037/0278-6133.22.3.270

[55] Jöreskog KG, Sörbom D (2006) LISREL 8.80 [Computer software]. Skokie, IL: Scientific Software International, Inc.

[56] Snijders TAB, Bosker RJ (1999) Multilevel Analysis: An Introduction to Basic and Advanced Multilevel Modeling. London: Sage Publishers.

[57] Heck RH, Thomas SL (2000) An introduction to multilevel modeling techniques. Mahwah, NJ: Lawrence Erlbaum Associates, Inc.

[58] Raudenbush SW, Bryk AS (2002) Hierarchical linear models: Applications and data analysis methods. Thousand Oaks: Sage Publications.

[59] Slovic P, Finucane ML, Peters E, MacGregor DG (2007) The affect heuristic. Eur J Oper Res 177:1333–1352.

